# Desiccation does not drastically increase the accessibility of exogenous DNA to nuclear genomes: evidence from the frequency of endosymbiotic DNA transfer

**DOI:** 10.1186/s12864-020-06865-8

**Published:** 2020-07-01

**Authors:** Xixi Li, Cheng Fang, Jun-Peng Zhao, Xiao-Yu Zhou, Zhihua Ni, Deng-Ke Niu

**Affiliations:** 1 MOE Key Laboratory for Biodiversity Science and Ecological Engineering and Beijing Key Laboratory of Gene Resource and Molecular Development, College of Life Sciences, Beijing Normal University, Beijing, 100875 China; 2 College of Life Sciences, Hebei University, Baoding, 071002 China

**Keywords:** Horizontal gene transfer (HGT), Nuclear mitochondrial DNA (NUMT), Nuclear plastid DNA (NUPT), Double-strand breaks (DSBs), Non-homologous end joining (NHEJ), Bdelloid rotifers

## Abstract

**Background:**

Although horizontal gene transfer (HGT) is a widely accepted force in the evolution of prokaryotic genomes, its role in the evolution of eukaryotic genomes remains hotly debated. Some bdelloid rotifers that are resistant to extreme desiccation and radiation undergo a very high level of HGT, whereas in another desiccation-resistant invertebrate, the tardigrade, the pattern does not exist. Overall, the DNA double-strand breaks (DSBs) induced by prolonged desiccation have been postulated to open a gateway to the nuclear genome for exogenous DNA integration and thus to facilitate the HGT process, thereby enhancing the rate of endosymbiotic DNA transfer (EDT).

**Results:**

We first surveyed the abundance of nuclear mitochondrial DNAs (NUMTs) and nuclear plastid DNAs (NUPTs) in five eukaryotes that are highly resistant to desiccation: the bdelloid rotifers *Adineta vaga* and *Adineta ricciae*, the tardigrade *Ramazzottius varieornatus*, and the resurrection plants *Dorcoceras hygrometricum* and *Selaginella tamariscina*. Excessive NUMTs or NUPTs were not detected. Furthermore, we compared 24 groups of desiccation-tolerant organisms with their relatively less desiccation-tolerant relatives but did not find a significant difference in NUMT/NUPT contents.

**Conclusions:**

Desiccation may induce DSBs, but it is unlikely to dramatically increase the frequency of exogenous sequence integration in most eukaryotes. The capture of exogenous DNA sequences is possible only when DSBs are repaired through a subtype of non-homologous end joining, named alternative end joining (alt-EJ). Due to the deleterious effects of the resulting insertion mutations, alt-EJ is less frequently initiated than other mechanisms.

## Background

Horizontal gene transfer (HGT, also termed lateral gene transfer) is the movement of genetic materials between organisms rather than from parent to offspring [1, 2]. HGT is well documented as the principal route of evolutionary innovation in bacteria and archaea, such as the acquisition of antibiotic resistance in pathogenic bacteria [3]. In contrast, the contribution of HGT to the evolution of eukaryotic genomes remains hotly debated [4–10]. An example of this controversy is the amount of human genes acquired via HGT [11]. In the first draft of the human genome, 223 proteins were found to be more similar to bacterial proteins than to any eukaryotic protein sequences available at that time, and these human proteins were suggested to have arisen from HGTs [12]. However, this result was quickly refuted by extensive analyses of the candidate genes [13, 14], and according to the opponents, gene loss in other eukaryotic lineages was the most likely explanation. Fourteen years later, Crisp et al. [15] confirmed 17 previously reported foreign genes and found 128 additional foreign genes in the human genome in a large-scale comparative study; however, their conclusion was refuted by a case-by-case re-analysis of the 145 genes [16]. Almost at the same time, another group claimed that they had discovered 1467 HGT regions in the human genome, involving 642 known genes [17]. We do not expect that there will be an end to this debate in the near future.

Compared with HGT, gene transfer from the organellar genome to the nuclear genome, termed endosymbiotic gene transfer (EGT), has been widely observed and well accepted in eukaryotes [2, 18, 19]. Indeed, mitochondria and plastids are thought to be of endosymbiotic origin, and most of their genetic sequences have been transferred to the nuclear genome. Although the effective transfer of functional protein-coding genes has slowed or even stopped in some lineages, the process of DNA fragment transfer from organelle to nucleus still actively occurs in most eukaryotic lineages [19–22]. Mitochondrial DNA segments recently inserted into nuclear genomes are termed nuclear mitochondrial DNAs (NUMTs), whereas plastid DNA segments recently inserted into nuclear genomes are termed nuclear plastid DNAs (NUPTs). Furthermore, NUMTs and NUPTs are collectively termed nuclear organelle DNA (norgDNA) [23].

Both HGT and EGT are special cases of DNA transfer. In most cases, the transferred DNA is non-functional in the acceptor nucleus and is merely junk DNA. DNA transfer provides raw materials for gene transfer. Only when the transferred DNA contains entire genes and the transferred genes have been fixed in the evolution of the nuclear genome can we regard it as a case of HGT or EGT. If most of the transferred DNAs have been eliminated in natural selection, environmental factors that affect horizontal DNA transfer (HDT) and endosymbiotic DNA transfer (EDT) would have a very weak influence on the frequency of HGT and EGT. Only when the effects of the transferred DNAs are slightly deleterious or neutral to the host could the environmental factors that affect HDT and EDT have significant effects on the frequency of HGT and EGT.

In most multicellular animals and plants, the opportunity to directly pass genetic information to future generations is restricted to a small proportion of cells, i.e., germ line cells [24], which may serve as a strong barrier to HGT and HDT. However, in both animals and plants, a few weakly protected unicellular or early developmental stages exist in which there are some opportunities for environmental DNA to enter germ cells and be inherited [25]. Distinctive animals in the field of HGT are bdelloid rotifers. Although the frequency of HGT depends on the evaluation method used, the quality of the sequenced genome, and the specific species studied, a pattern of extensive HGT in bdelloid rotifers has consistently been reported [26–30]. Indeed, HGTs in bdelloid rotifers are so frequent that some researchers have suggested that this process can act as an alternate form of sex to facilitate the genetic exchange of rotifer DNA among these organisms [31]. Although evidence for genetic exchanges among bdelloid rotifers has been shown to be driven by cross-contamination in a recent paper [32], there is to our knowledge no controversy regarding the extensive pattern of HGT from non-metazoan species to bdelloid rotifers. Bdelloid rotifers are tiny invertebrates that live in ephemeral aquatic habitats and therefore frequently experience severe desiccation. When facing desiccation, bdelloid rotifers enter a form of dormancy called anhydrobiosis [33, 34], and the prolonged desiccation of the anhydrobiotic bdelloid rotifer *Adineta vaga* induces DNA double-strand breaks (DSBs) [35]. Such DSBs, which were vividly described as the “gateway to genetic exchange” by Hespeels et al. [35], are often believed to be the main cause of the high frequency of HGT in bdelloid rotifers [26–28, 30, 31, 35, 36]. The observed frequency of gene transfer in bdelloid rotifers has generally been attributed to the convenience of the transfer process rather than the fixation rate of the transferred genes. In this context, gene transfer has almost the same meaning as DNA transfer.

In addition to bdelloid rotifers, a number of other organisms also enter an anhydrobiotic state when their environment becomes dry. Another striking example is the phylum Tardigrada [34], in which prolonged desiccation has been shown to induce a small amount of DSBs [37]. Frequent HGT is naturally expected to occur in tardigrade species, by analogy with bdelloid rotifers, via a similar mechanism. Although no further support for the gateway hypothesis has been revealed by studies on tardigrades, the controversy of HGT in eukaryotes has been amplified. The first analysis of a tardigrade draft genome (*Hypsibius exemplaris*, originally misidentified with the congeneric *H. dujardini* [38]) suggested that HGT occurred more extensively in tardigrades than in bdelloid rotifers; 17.5% of its genes were identified as foreign and originating from bacteria, plants, fungi, and archaea [39]. However, subsequent independent sequencing of the same species and another tardigrade species, *Ramazzottius varieornatus*, consistently showed a much lower percentage of foreign genes in tardigrades than the prior study, with 2.3% reported as the upper bound [40–45]. As most previously identified foreign genes were found to be the result of DNA contamination from non-target organisms, tardigrades should not be regarded as a special group of animals with the high frequency of HGT [44]. Overall, the predominant objections to the claimed extensive HGT in tardigrades reject the gateway hypothesis and question whether prolonged desiccation truly facilitates HDT by inducing DSBs in the nuclear genome.

In this paper, we evaluated whether desiccation facilitates DNA transfer and provides increased raw materials for the fixation of gene transfer in evolution. Because donor sequences are readily available, EDT events are more conveniently identified than HDT events. From entering the nucleus to integrating into the nucleus genome, EDT and HDT processes share the same essential steps. For this reason, the environmental or physiological factors that affect the frequency of EDT likely have the same effect on HDT. Therefore, the effects of desiccation on EDT would provide direct evidence for the effects of desiccation on HDT, which, consequently, has implications for the effect of desiccation on the frequency of HGT when the frequency of HGT is assumed to be largely determined by HDT, as in bdelloid rotifers and tardigrades [26–28, 31, 35, 36, 39]. In this study, we sought to gain new insight into the debates on the effects of desiccation on HGT by analysing the abundance of NUMTs and NUPTs in eukaryotic organisms that frequently experience desiccation.

## Results

### A trade-off between genome quality and sample size

In the NCBI genome database [46], the assembly levels of nuclear genomes are divided into four categories: complete genome, chromosome, scaffold, and contig. For a trade-off between genome quality and sample size, we evaluated the effects of genome assembly level on the detected abundances of NUMTs/NUPTs using a phylogenetic comparative method, phylogenetic generalized least squares (PGLS). The contig level was designated 1, and the other three levels (scaffold, chromosome, and complete genome) were designated 2; the PGLS regression subsequently revealed that the assembly level was significantly correlated with the number and total length of NUPTs (*p* < 0.05 for both cases) and the number of NUMTs (*p* = 0.037) but not the total length of NUMTs (Table 1). These results indicate that the abundances of NUMTs/NUPTs might be overestimated or underestimated when the analysed nuclear genomes are assembled at the contig level. Furthermore, we performed PGLS regression between NUMT/NUPT abundance and assembly level by assigning the scaffold level as 3 and the other two levels (chromosome and complete genome) as 4 (Table 1). The number of NUPTs was also significantly correlated with genome assembly level (*p* = 0.044). However, no significant correlations were detected between the assembly level and the number of NUMTs or the total length of NUMTs or NUPTs (Table 1). The scaffold level might also distort the estimation of the abundance of NUPTs, but the effects at the scaffold level are much weaker than those at the contig level. With a trade-off between the sample size and the accuracy of the estimation, we retained all of the nuclear genomes assembled at the scaffold, chromosome, and complete genome levels.

**Table 1:** Correlations between nuclear genome assembly level and the abundance of NUMTs/NUPTs

	NUMT	NUPT
Number of species		
Contig (1)	16	8
Scaffold/chromosome/complete genome (2)	216	106
Number of NUMTs/NUPTs		
λ	0.983	0.401
R^2^	0.019	0.150
Slope	−0.713	1.393
*p*	0.037	2.1 × 10^−5^
Total length of NUMTs/NUPTs		
λ	0.942	0.718
R^2^	0.008	0.046
Slope	−0.468	0.807
*p*	0.185	0.022
Number of species		
Scaffold (3)	151	56
Chromosome/complete genome (4)	65	50
Number of NUMTs/NUPTs		
λ	0.985	0.507
R^2^	4 × 10^−4^	0.038
Slope	0.050	0.346
*p*	0.769	0.044
Total length of NUMTs/NUPTs		
λ	0.935	0.745
R^2^	0.004	0.015
Slope	−0.187	0.239
*p*	0.346	0.218

For one plant for which the data of both NUMTs and NUPTs were available, we regarded it as two samples, one NUMT sample and one NUPT sample, rather than combining them into one norgDNA sample. The correlations were analysed by phylogenetic generalized least squares (PGLS) regression. The numbers in the parentheses after the genome assembly levels are the numbers that were assigned to the assembly levels in the PGLS regression analysis. λ is the phylogenetic signal. The approach of this value to one necessitates the use of phylogenetic comparative methods, such as PGLS

### The most desiccation-tolerant organisms do not have excessive NUMTs/NUPTs

Water deficiency is one of the most common abiotic stress factors for organisms living on land, and terrestrial organisms have evolved two solutions to cope with environmental drying [47]. The first is to conserve water and avoid severe body water deprivation, such as waxy coatings on plant shoots and the protective cocoon of the African lungfish, *Protopterus annectens* [48]. The second solution is to tolerate body water loss. The term anhydrobiosis is often used for the almost completely dehydrated but viable state of an organism experiencing extreme desiccation [49, 50]. Desiccation tolerance exists on a scale where the most desiccation-tolerant organisms are those that can enter anhydrobiosis and thus survive desiccation at any stage of their life cycle. The commonly studied organisms of this group are bdelloid rotifers, tardigrades, and resurrection plants [51–53].

DSBs induced by prolonged desiccation have been suggested to open the “gateway to genetic exchange” and account for the high frequency of HGT in bdelloid rotifers [26–28, 30, 31, 35]. However, prolonged desiccation in tardigrades does not result in an elevated level of HGT [44]. Using EDT as a proxy for HDT, we tested whether prolonged desiccation enhanced HDT in these desiccation-tolerant invertebrates. We detected 22 and 4 NUMTs, with total lengths of 32 kb and 14 kb, in the genomes of the desiccation-tolerant bdelloid rotifers *A. vaga* and *A. ricciae*, respectively. Similarly, in the genome of the desiccation-tolerant tardigrade *R. varieornatus*, we found 53 NUMTs, with a total length of 33 kb. If exceptionally high HGT levels in bdelloid rotifers are attributed to the anticipated high accessibility of their nuclear genomes to exogenous DNA, the NUMT content in bdelloid rotifers should also be exceptionally high compared with that in other invertebrates. In invertebrates, the NUMT density in honeybee *Apis mellifera* [54] is considered “exceptionally high”. To reduce methodological artefacts, we re-surveyed the honeybee genome with our parameters and detected 1791 NUMTs, with a total length of 724 kb. We also surveyed the NUMT abundances of other invertebrates and confirmed that the NUMT contents of bdelloid rotifers and tardigrades are not exceptionally high (Table 2). The NUMT contents of the desiccation-tolerant bdelloid rotifers and tardigrades are similar to the median NUMT content of other invertebrates but are 13–185 times lower than the average NUMT number of other invertebrates and are 2–6 times lower than the average value of the total NUMT length in other invertebrates (Table 2). The high level of HGT in desiccation-tolerant bdelloid rotifers is not accompanied by a high frequency of EDT.

**Table 2:** The norgDNA contents of invertebrates and plants

	Type	Number of species	Number of norgDNAs	Total length of norgDNAs (kb)
*Adineta vaga*	NUMT		22	32
*A. ricciae*	NUMT		4	14
*Ramazzottius varieornatus*	NUMT		53	33
Other invertebrates	NUMT	193	742 ± 7810 (32)	97 ± 781 (15)
*Dorcoceras hygrometricum*	NUMT		3696	1232
	NUPT		1610	467
*Selaginella tamariscina*	NUPT		637	520
Other plants	NUMT	67	9309 ± 26,177 (4182)	2735 ± 9044 (895)
	NUPT	118	3015 ± 3907 (2014)	940 ± 1280 (509)

The values of other invertebrates and other plants presented in this table are mean value ± S. D. (median value)

Resurrection plants can survive extreme desiccation and maintain a quiescent state for months to years [55]. To examine the relationship between prolonged desiccation and the frequency of DNA transfer, we also surveyed the abundance of NUMTs/NUPTs in these plants. In the genome of the flowering plant *Dorcoceras hygrometricum*, we detected 3696 NUMTs with a total length of 1232 kb and 1610 NUPTs with a total length of 467 kb. In the genome of the spike moss *Selaginella tamariscina*, we detected 637 NUPTs with a total length of 520 kb. We did not obtain the mitochondrial genome sequences of *S. tamariscina* and consequently did not obtain any results regarding its NUMT content. The NUMT content of the resurrection plant *D. hygrometricum* was much higher than the NUMT contents of the desiccation-tolerant bdelloid rotifers and tardigrades (Table 2). Nonetheless, previous studies, together with our own survey, have shown that plant genomes generally have much higher NUMT contents than invertebrates [20, 21]. The norgDNA contents we detected in the resurrection plants were lower than the average and median values of the norgDNA contents in other plants except that the total length of NUPTs in *S. tamariscina* was a little longer than the median value of the lengths of NUPTs in other plants (520 vs. 509 kb, Table 2).

### Pairwise comparison of organisms differing in desiccation tolerance

In the second grade of desiccation tolerance, anhydrobiosis is restricted to particular developmental stages, such as dormant eggs of the water flea *Daphnia*, cysts of primitive crustaceans such as the brine shrimp *Artemia salina*, and orthodox seeds of most angiosperms [56, 57]. In the last grade, all stages of the life cycle are desiccation sensitive, including the eggs of animals, recalcitrant seeds, and the embryos of viviparous plants. From this perspective, a viviparous plant or a plant with recalcitrant seeds is more sensitive to desiccation than a plant with orthodox seeds.

**Fig. 1:**
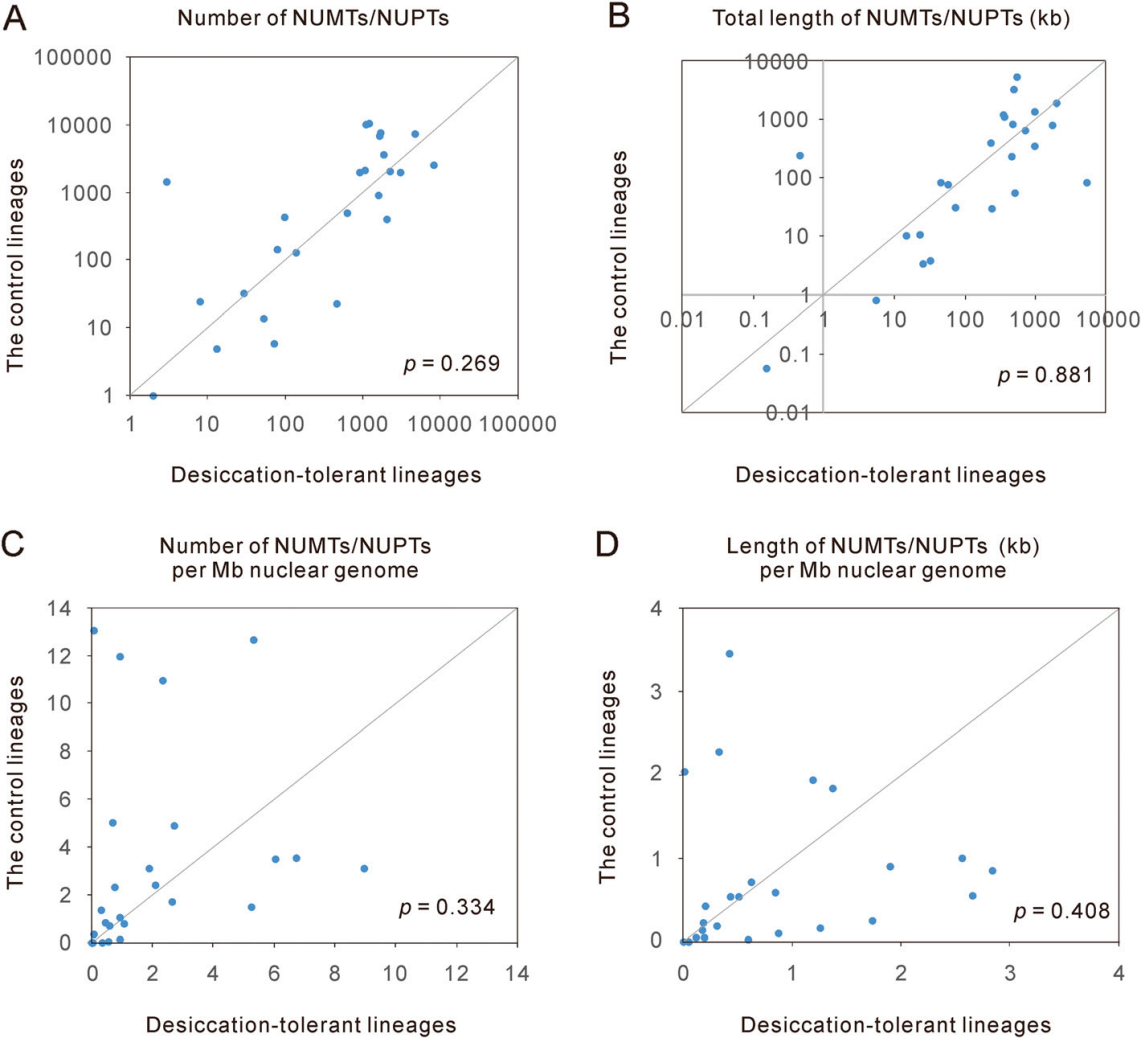
Comparison of the NUMT/NUPT contents between desiccation-tolerant organisms and their relatively less desiccation-tolerant relatives. The NUMT/NUPT contents were measured by the number of NUMTs/NUPTs (**a**), the total length of NUMTs/NUPTs (**b**), the number of NUMTs/NUPTs per Mb nuclear genome (**c**), and the total length of NUMTs/NUPTs per Mb nuclear genome (**d**). The diagonal line represents cases in which desiccation-tolerant organisms and their paired relatives have the same NUMT/NUPT contents. Points above the line represent cases in which desiccation-tolerant organisms have lower NUMT/NUPT contents than their paired relatives, while points below the line indicate the reverse. All the *p* values were calculated using two-tailed Wilcoxon signed-rank tests. The exact values of the NUMT/NUPT contents are shown in Additional file 1: Table S5

To reach a general conclusion regarding the relationship between desiccation and the frequency of EDT, we compared 24 pairs of lineages for which each pair of lineages differed in desiccation tolerance (Table 3). The closest relatives used as controls for desiccation-tolerant organisms were selected from widely used phylogenetic databases, including Timetree, NCBI taxonomy, and the Angiosperm Phylogeny Website [96–98]. In cases where one phylogenetic branch contained two desiccation-tolerant species or two control species, we used the average value of the NUMT/NUPT contents of the two species. For example, the average value of desiccation-tolerant bdelloid rotifers *A. vaga* and *A. ricciae* was used to compare the average value of desiccation-sensitive bdelloid rotifers *R. magnacalcarata* and *R. macrura*. Nonparametric pairwise comparison did not reveal significant differences between desiccation-tolerant lineages and their controls in either the number of NUMTs/NUPTs (Wilcoxon signed-ranks test, *P* = 0.269, Fig. 1a) or the total length of NUMTs/NUPTs (Wilcoxon signed-ranks test, *P* = 0.881, Fig. 1b). Overall, a large nuclear genome is expected to have additional sites for NUMT/NUPT integration and is thus likely to contain additional NUMTs/NUPTs. To control for the influence of nuclear genome size, we compared the density of NUMTs/NUPTs in nuclear genomes and still found no significant differences between desiccation-tolerant lineages and their controls (Fig. 1c, d). These pairwise comparisons were also performed within invertebrates and within plants separately. Nevertheless, no significant differences were observed (Wilcoxon signed-ranks test, *P* > 0.10 for all the cases). Furthermore, we separated the 24 pairs into two groups according to the average values of the nuclear genome size of each pair. No significant differences were observed in either organisms with large nuclear genomes or organisms with small nuclear genomes (Wilcoxon signed-ranks test, *P* > 0.10 for all the cases).

**Table 3:** Desiccation-tolerant organisms and their controls used in this study

	Desiccation-tolerant species	Control
Invertebrates		
Bdelloidea	*Adineta ricciae* and *A. vaga* [29]	*Rotaria macrura* and *R. magnacalcarata* [29]
Hypsibioidea	*Ramazzottius varieornatus* [44]	*Hypsibius dujardini* [44, 58]
Culicidae	*Aedes aegypti* [59, 60]	*Culex quinquefasciatus* [61]
*Anopheles*	*Anopheles culicifacies* [62]	*Anopheles funestus* [63]
*Dermatophagoides*	*Dermatophagoides farina* [64]	*Dermatophagoides pteronyssinus* [64]
*Meloidogyne*	*Meloidogyne javanica* [65]	*Meloidogyne incognita* [65]
*Polypedilum*	*Polypedilum vanderplanki* [66]	*Polypedilum nubifer* [66]
*Steinernema*	*Steinernema carpocapsae* [67]	*Steinernema glaseri* [67]
Plants		
Pooideae	*Aegilops tauschii* [68]	*Lolium perenne* [69]
Fagales	*Betula nana* [70]	*Juglans regia* [71]
Malvaceae	*Corchorus capsularis* [70]	*Theobroma cacao* [72]
Pentapetalae	*Dorcoceras hygrometricum* [73]	*Fagopyrum esculentum* [74]
Malvoidae	*Hibiscus syriacus* [75]	*Gossypium hirsutum* [76]
Crotonoideae	*Manihot esculenta* [77]	*Hevea brasiliensis* [78]
Oryzeae	*Oryza sativa Japonica Group* [70]	*Zizania latifolia* [79]
Ericales	*Primula veris* [80]	*Actinidia chinensis* [81]
*Selaginella*	*Selaginella tamariscina* [82]	*Selaginella moellendorffii* [82]
Caryophyllales	*Silene latifolia* [83]	*Spinacia oleracea* [84]
*Solanum*	*Solanum commersonii* [85]	*Solanum tuberosum* [86]
*Solanum*	*Solanum pennellii* [87]	*Solanum melongena* [88]
Andropogonodae	*Sorghum bicolor* [89]	*Zea mays* [90]
Fungi		
Onygenales	*Coccidioides immitis* [91]	*Paracoccidioides brasiliensis* [92]
Hypocreales	*Fusarium graminearum* [93]	*Ophiocordyceps sinensis* [94]
*Pseudogymnoascus*	*Pseudogymnoascus pannorum* [95]	*Pseudogymnoascus destructans* [95]

The particular words or sentences supporting the desiccation tolerance/sensitivity of the above species were extracted from the references and are shown in Additional file 1: Table S3

Because insertions of norgDNAs into coding regions would disrupt gene function, a compact nuclear genome is a less accessible acceptor than a nuclear genome with a high percentage of noncoding sequences. Therefore, it was necessary to check whether the paired genomes had similar levels of compactness. From the NCBI genome database [46], the Ensembl Genomes database (Release 41) [99], and the Ensembl database (Release 94) [100], we got the annotation files of coding regions for six pairs of species. Pairwise comparison did not reveal a significant difference in genome compactness (Wilcoxon signed-ranks test, *P* = 0.293). As the NUMTs and NUPTs of *Sorghum bicolor* and *Zea mays* were regarded as independent samples, these six pairs of species have seven pairs of NUMT/NUPT data. The Wilcoxon signed-ranks test of these seven pairs did not reveal any significant differences in the abundances of NUMTs/NUPTs (*P* > 0.10).

Although most EDTs occur via direct transfer of organellar DNA to the nucleus, there is also evidence supporting EDT mediated by RNA molecules [101]. Therefore, we also compared the NUMTs/NUPTs contributed by the transcribed regions of organellar genomes, i.e., protein-coding genes, tRNA, and rRNA. Similar to the above results, no significant difference was detected between desiccation-tolerant species and their controls (Wilcoxon signed-ranks test, *P* > 0.10 for all cases, Additional file 1: Table S1).

## Discussion

Desiccation is a widely observed cause of DSB formation in bacteria, plants and animals, including bdelloid rotifers [33–35, 102–110]. Organisms that frequently encounter desiccation stress have evolved efficient mechanisms to repair DSBs [111]. Accordingly, the radioresistance of *Deinococcus radiodurans* and bdelloid rotifers has been attributed to their adaptation to desiccation stress [102, 112]. As the DSBs in nuclear genomes are opportunities for the integration of exogenous DNA, DSBs have been postulated to be a gateway for HGT [35]. If the DSBs of desiccation-tolerant organisms serve as an efficient gateway for exogenous DNA integration, the frequency of EDT should also be dramatically increased by desiccation. However, we did not observe excessive NUMTs/NUPTs in the genomes of the desiccation-tolerant organisms, the bdelloid rotifers *A. vaga* and *A. ricciae*, the tardigrade *R. varieornatus*, or the resurrection plants *D. hygrometricum* or *S. tamariscina*. Further pairwise comparison of 24 desiccation-tolerant lineages with their controls also did not reveal evidence for desiccation-elevated EDT frequency.

Notably, there are also a few exceptions to the general observations that desiccation could induce DSBs. In the anhydrobiotic tardigrade *Milnesium tardigradum*, the process of desiccation also induces DNA damage but at only minor levels [37]. In the tardigrade *R. varieornatus*, a protein that suppresses DNA breaks has been identified [45]. In some bacteria, *Nostoc commune*, *Chroococcidiopsis* sp. CCMEE 029 and possibly *Bacillus pumilus*, no evidence of extensive genome fragmentation was observed in cells following prolonged desiccation [107, 113, 114]. Some desiccation-tolerant eukaryotes might lack an elevated EDT frequency because they happen to be such exceptions in which desiccation does not induce DSBs. Furthermore, even in organisms in which desiccation does induce DSBs, frequent DSBs do not necessarily increase the frequency of exogenous DNA integration into nuclear genomes. Indeed, the integration of exogenous DNA, i.e., HDT and EDT, depends on the specific mechanisms of DSB repair, for which there are two main categories: homologous recombination (HR) and non-homologous end joining (NHEJ). The capture of exogenous DNA sequences is possible only when DSBs are repaired through a subtype of NHEJ, named alternative end joining (alt-EJ), which is initiated much less frequently than other mechanisms [115–117]. The choice between the HR and NHEJ repair pathways and among different subtypes of NHEJ depends on phylogenetic position, cell cycle phase and repetitive sequence content [118–120]. If an organism predominantly uses HR for DSB repair, desiccation-induced DSBs do not open a gateway for HDT and EDT. In general, organisms with compact genomes, such as yeasts, preferentially use HR; in contrast, NHEJ is the predominant DSB repair pathway in mammals and plants with large and complex genomes. We did not observe significant differences between desiccation-tolerant plants and their controls. In addition, significant differences were not detected in organisms with relatively larger nuclear genomes. Exceptions to the general rule between genome size and HR/NHEJ usage, bdelloids have more copies of gene involved in NHEJ than other animals and thus preferentially use NHEJ in their DSB repairs [121]. However, we did not observed a higher NUMT content in bdelloids than other invertebrates.

If desiccation-induced DSBs open the gateway for exogenous DNA but the availability of organellar DNA fragments is under strong constraints, the frequency of NUMTs/NUPTs would not be elevated by desiccation. Organisms such as *Ostreococcus tauri* have a single plastid or mitochondrion in each cell. The lysis of these organelles would almost certainly result in cell death. These organisms, with a limited availability of donor organellar DNA, have been shown to have much lower amounts of NUMTs or NUPTs than organisms with multiple organelles per cell [122]. However, all of the organisms we studied have multiple organelles per cell, and the lysis of organelles and release of organellar DNA fragments are not under strong constraints during desiccation.

As NUMTs/NUPTs are insertion mutations, their abundance and the frequency of EGT/HGT depend not only on the insertion rate but also on the fixation possibility during evolution [123]. For a compact nuclear genome with a limited amount of junk DNA sequences, most insertions would disrupt the nuclear genetic information and thus be selected against. Similar to previous reports [20, 122, 124], we observed a significant positive correlation between the abundance of NUMTs/NUPTs and nuclear genome size in our dataset (Spearman’s rho = 0.737 and 0.803 for NUMT/NUPT number and total length, respectively, and *P* < 0.01 for both cases).

It should be noted that the diversity of environmental DNA sequences is theoretically unlimited, significantly larger than that of the organellar DNA sequences. Meanwhile, for most eukaryotes, the copy number of the organellar DNA sequences in a cell is larger than most environmental DNA sequences that are in proximity to the cell. We are not interested in comparing the absolute amount of NUMT/NUPT with the nuclear DNA sequences obtained recently from environments. Based on the assumption is that environmental DNA and organellar DNA share the same mechanisms from entering the nucleus to the integration into the nuclear DNA, we proposed that a eukaryote with a higher NUMT/NUPT content should acquire more environmental DNA than other eukaryotes.

Our study was based on the assumption that the frequency of EDT could be used as a proxy for the frequency of HDT, which in turn is probably correlated with the frequency of HGT. This assumption would be invalidated if organellar DNAs could not exit organellar membranes, while environmental DNAs could cross the plasma membrane during desiccation. From the properties of protectants (intrinsically disordered proteins, trehalose, etc.) that can prevent the loss of membrane integrity [125–128], the preferential protection of organellar membranes were not expected. Moore et al. [129] suggested that intrinsically disordered proteins could offset the damage during the desiccation of liposomes that mimic the lipid compositions of the mitochondrial membrane and the plasma membrane. More importantly, they found that liposome stabilization by intrinsically disordered proteins was not dependent on the lipid composition. Thus, there is no evidence for the preferential protection of the mitochondrial membrane over the plasma membrane or the plasma membrane over the mitochondrial membrane.

In this study, we examined whether desiccation facilitates the integration of exogenous DNA into nuclear genomes. In principle, desiccation could also induce DSBs in mitochondrial and plastid genomes. It will be interesting to determine whether desiccation can facilitate the transfer of exogenous DNA segments (including both nuclear DNA and DNA from the environment) into organellar genomes. Mitochondrial and plastid DNA of nuclear origin are termed MINC and PLNC, respectively [130]. With references to the organellar/nuclear genes or nuclear retrotransposons, some MINCs and PLNCs could be differentiated from NUMTs and NUPTs. Compared with plastid genomes and animal mitochondrial genomes, plant mitochondrial genomes are unique in their excessive size relative to their coding capacity [131]. The significant amount of noncoding DNA in plant mitochondrial genomes makes it difficult to differentiate NUMTs and MINCs in most shared nuclear–mitochondrial segments [130]. As far as we know, the shared nuclear–mitochondrial segments are simply regarded as NUMTs in all previous genome-wide studies of NUMTs [20, 21, 54, 122, 132–134]. In the present study, we adopted the same approximation. We suggest that MINCs are significantly less frequent than NUMTs; therefore, such approximations are unlikely to affect the conclusions qualitatively. This conjecture was inspired by the paucity of NUMTs/NUPTs in organisms that have only one mitochondrion or one plastid per cell [122, 132]. The lysis of a single mitochondrion or plastid would almost certainly result in cell death; therefore, mono-mitochondria and monoplastids restrict the production of norgDNAs. With the same logic, a single nucleus also restricts the formation of MINCs and PLNCs because the lysis of the nucleus and release of nuclear DNA segments into the cytoplasm would result in cell death. Nevertheless, we hope to distinguish NUMTs and MINCs accurately in the future once additional unique plant mitochondrial sequences are identified [135].

## Conclusions

In this study, we did not find evidence that desiccation significantly increased the norgDNA content. The results are inconsistent with the hypothesis that prolonged desiccation could open a gateway to the nuclear genome for exogenous DNA integration. Therefore, the prolonged desiccation experienced by bdelloid rotifers, tardigrades, and resurrection plants likely do not provide additional raw materials (i.e., HDTs) for HGT. However, notably, the fate of HDTs might be entirely different from that of EDTs. After a long evolutionary history, most organellar genes have been transferred to nuclear genomes, and only tens to a few hundreds of protein-coding genes are retained in organellar genomes. There is an upper limit of further successful EGT. In contrast, there is no such an upper limit for HGT. Any DNA sequences acquired from other species or even other phylogenetic domains that could help the host survive under severe stresses, such as desiccation, will be favoured in natural selection and fixed in the host nuclear genome. Therefore, our results should not be regarded as evidence against frequent HGT in bdelloid rotifers and tardigrades. Readers interested in this subject should refer to recent studies on HGT, such as [29, 44].

## Methods

From the widely used genome databases [46, 99, 136], we first retrieved a list of eukaryotes whose nuclear genomes and at least one organellar genome (mitochondrion or plastid) had been sequenced. To avoid interference from the effects of a single plastid or single mitochondrion per cell on the abundance of NUMTs or NUPTs [122], protists were not included in our study. As vertebrates generally use the strategy of avoiding severe body water deprivation to survive in water deficient environments rather than mechanisms to adapt cellular dehydration [48], they are not suitable materials to examine the effects of desiccation on organelle DNA accumulation in nuclear genomes.

### Selection of desiccation-tolerant species and their controls

Then, we searched the desiccation-tolerant species and their controls using the Latin names of the above species together with desiccation tolerant, drought tolerant, desiccation sensitive, drought sensitive, desiccation resistant, drought resistant, orthodox seed, recalcitrant seed, viviparous plant, resurrection plant, xerophyte, xerotolerance, xerophilic, and anhydrobiosis as keywords in Google [137] and Google Scholar [138]. In addition, we retrieved a species list of the recalcitrant and orthodox seeds from the Seed Information Database [70]. Plants with recalcitrant seeds and viviparous plants do not have a desiccation-tolerant seed stage in their life cycle. These plants can be regarded as more sensitive to desiccation than other plants with dry seeds, i.e., orthodox seeds. For each desiccation-tolerant lineage, we selected the most closely related and relatively less tolerant lineage to pair with it. The phylogenetic relatedness was determined using three phylogenetic databases: Timetree, NCBI taxonomy, and the Angiosperm Phylogeny Website [96–98]. Each lineage contained one or two species from a genus.

Although tardigrades are often regarded as models of desiccation-tolerant organisms, clear differences in desiccation tolerance have been demonstrated among them. The species *H. dujardini* is a poor anhydrobiote compared with other tardigrades [44, 58]. Therefore, *H. dujardini* was used as the control of the desiccation-tolerant tardigrade *R. varieornatus*.

During the above literature searching, we also found some desiccation-tolerant species (and/or their controls) whose mitochondrial genome sequences were not available in public databases. For these species, we acquired four mitochondrial genome sequences (*A. vaga*, *A. ricciae*, *R. magnacalcarata*, and *R. macrura*) from the authors of the reference [29]. Then, we assembled the mitochondrial genome sequences for one invertebrate, six fungi and eight plants using NOVOPlasty [139] and for another invertebrate (*Polypedilum nubifer*) using Geneious Prime (2019.2.1). The IDs of the whole-genome sequencing datasets, the mitochondrial genome sequence seeds, the plastid genome sequences, and the mitochondrial genomes used as references in assembling the mitochondrial genomes are shown in Additional file 1: Table S2. The mitochondrial genome sequences that we assembled are shown in Additional file 2: Data S1.

Finally, we obtained 24 pairs of desiccation-tolerant vs. control lineages (Table 3 and Additional file 1: Table S3).

### Detection and filtering of NUMTs and NUPTs

In addition to the mitochondrial genome sequences mentioned above, we downloaded other organellar genome sequences from the NCBI nucleotide database [136]. The nuclear genome sequences were downloaded from the NCBI genome database [46], the Ensembl Genomes database (Release 41) [99], and the Ensembl database (Release 94) [100]. The assembly accession number and exact source of each genome are shown in Additional file 1: Table S4.

The NUMTs and NUPTs of some model species have been well explored [20, 21, 122, 133, 134]. Because the different parameters used in previous studies and different levels of genome assembly would likely lead to discrepancies in NUPT/NUMT abundance [21, 122], we performed our own detection using the same set of parameters and with the expectation that over- or underestimation affects all species consistently. Organellar genome sequences were used as the queries to search against nuclear genomes using blastn (version 2.4.0+) with a threshold E value of 0.0001, a word size of 11, a match score of 2, a mismatch score of − 3, and gap cost values of 5 (existence) and 2 (extension), as described by Smith et al. [122]. To reduce false-positive hits resulting from the contamination of organellar sequences in nuclear genome sequences, short nuclear contigs that completely matched to organellar genome sequences were discarded. Redundancy in the blastn results was eliminated as formulated below. M_1_ and M_2_ were assumed to be two sequences residing in different positions of the same mitochondrial genome, and four nuclear sequences (N_1_, N_2_, N_3_, and N_4_) were assumed to independently reside in four different chromosomes. In the blastn results, M_1_ was matched to N_1_ and N_2_, whereas M_2_ was matched to N_2_, N_3_, and N_4_. Both M_1_ and M_2_ might be the parent sequence of N_2_. However, N_2_ originated from either M_1_ or M_2_. Therefore, N_2_ was found twice but should be counted only once in the calculation of the NUMT content. In total, the two mitochondrial sequences produced four NUMTs. The distribution of the identities between the NUMTs/NUPTs and their parent organellar sequences for all species presented in Table 3 are shown in Additional file 3: Fig. S1.

We also separately detected the NUMTs and NUPTs contributed by organellar sequences with known functions, including protein-coding genes, rRNA genes, and tRNA genes, using the above method and parameters.

## Supplementary information

**Additional file 1: Table S1.** The NUMT/NUPT contents (contributed by organellar transcribed sequences) of desiccation-tolerant species and their controls. **Table S2.** The information used in assembling mitochondrial genomes. **Table S3.** The information used in assigning desiccation-tolerant species and their controls. **Table S4.** Genome information of the eukaryotes used in this study. **Table S5.** The NUMT/NUPT contents (contributed by organellar genomes) of desiccation-tolerant species and their controls.

**Additional file 2: Data S1.** The mitochondrial genome sequences assembled in this study.

**Additional file 3: Figure S1.** The distribution of the identities between NUMTs/NUPTs and their parental organellar sequences.

## Data Availability

The datasets and sequences used in assembling the mitochondrial genomes and their sources are shown in Additional file 1: Table S2. The particular words or sentences used in assigning desiccation-tolerant species and their controls as well as their sources are shown in Additional file 1: Table S3. The accession number/assembly name and exact source of each genome used in counting NUMTs/NUPTs are shown in Additional file 1: Table S4. The mitochondrial genome sequences that we assembled in this study are shown in Additional file 2: Data S1.

## Abbreviations

HGT: Horizontal gene transfer
HDT: Horizontal DNA transfer
EGT: Endosymbiotic gene transfer
EDT: Endosymbiotic DNA transfer
NUMT: Nuclear mitochondrial DNA
NUPT: Nuclear plastid DNA
MINC: Mitochondrial DNA of nuclear origin
PLNC: Plastid DNA of nuclear origin
norgDNA: Nuclear organelle DNA
DSB: Double-strand break
NHEJ: Non-homologous end joining
alt-EJ: Alternative end joining
HR: Homologous recombination
PGLS: Phylogenetic generalized least squares
