# Supplementary material for: Desiccation does not drastically increase the accessibility of exogenous DNA to nuclear genomes: evidence from the frequency of endosymbiotic DNA transfer
Source: BMC Genomics. 2020 Jul 1;21:452. doi: 10.1186/s12864-020-06865-8 (PMC7329468; doi:10.1186/s12864-020-06865-8)
Supplement: Supplementary file 3 — Additional file 3: Figure S1. The distribution of the identities between NUMTs/NUPTs and their parental organellar sequences. [file 12864_2020_6865_MOESM3_ESM.pdf]

Figure S1 The distribution of the identities
between NUMTs/NUPTs and their parental organellar sequences

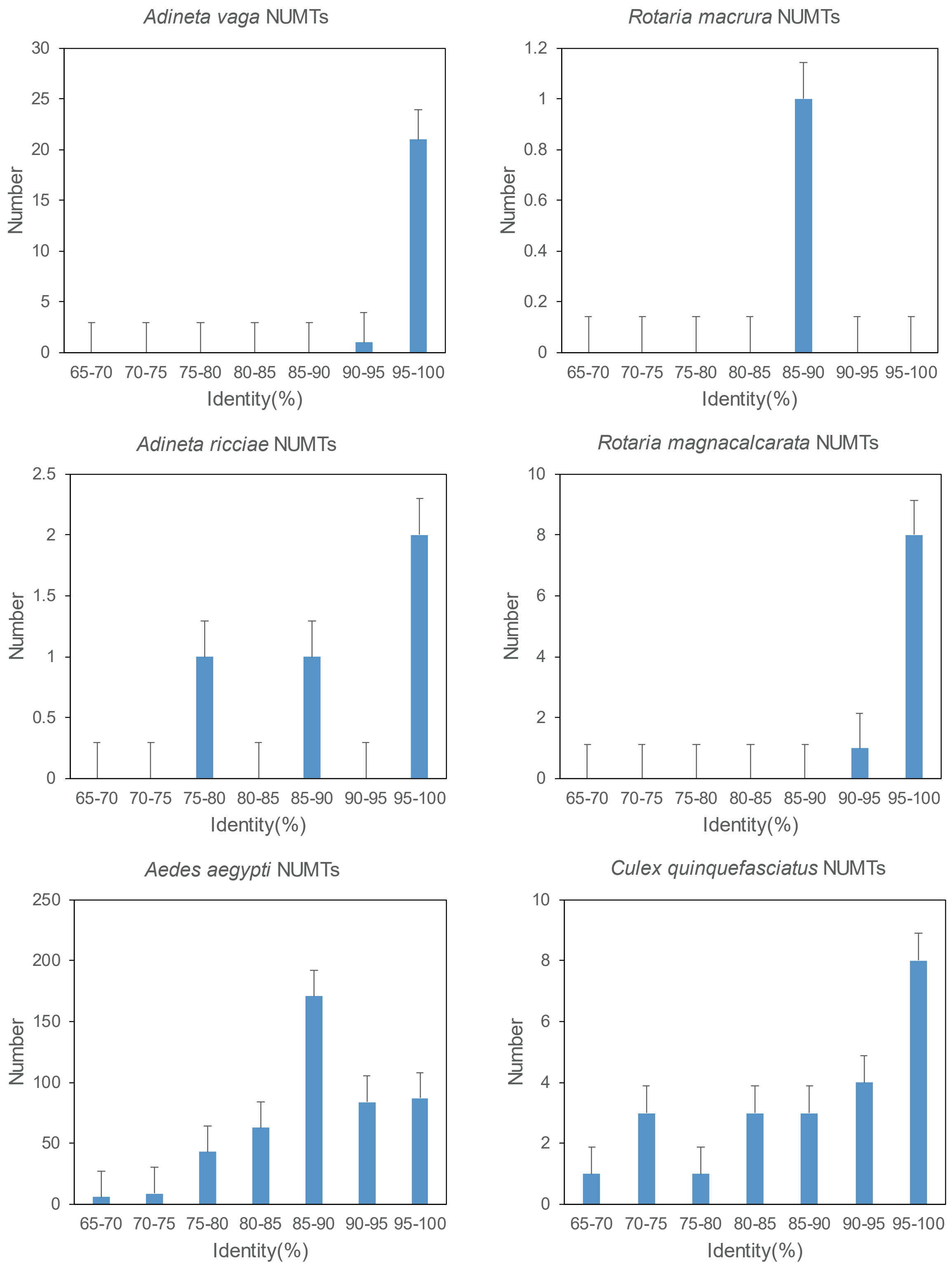

Continued on next page.

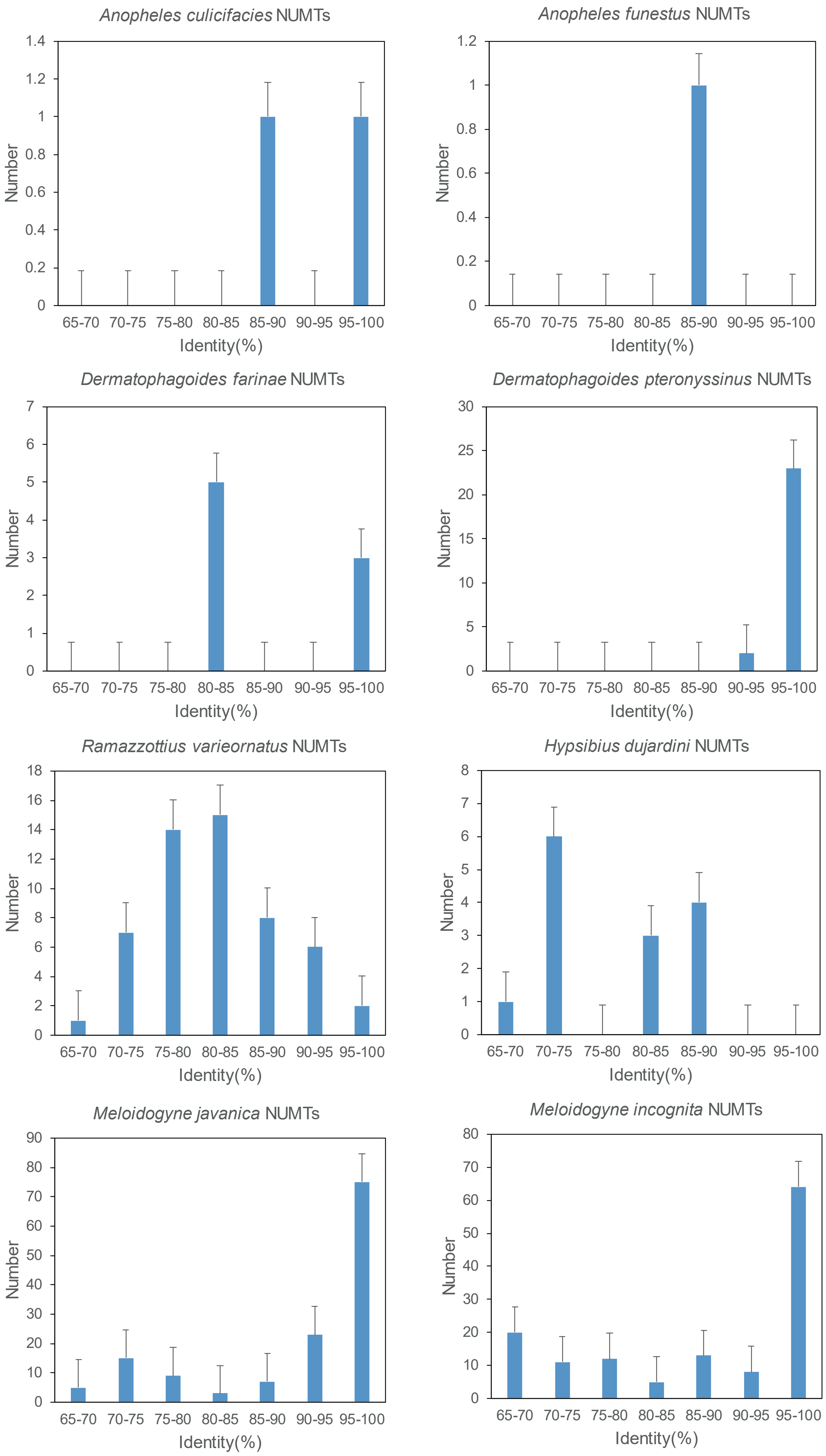

Continued on next page.

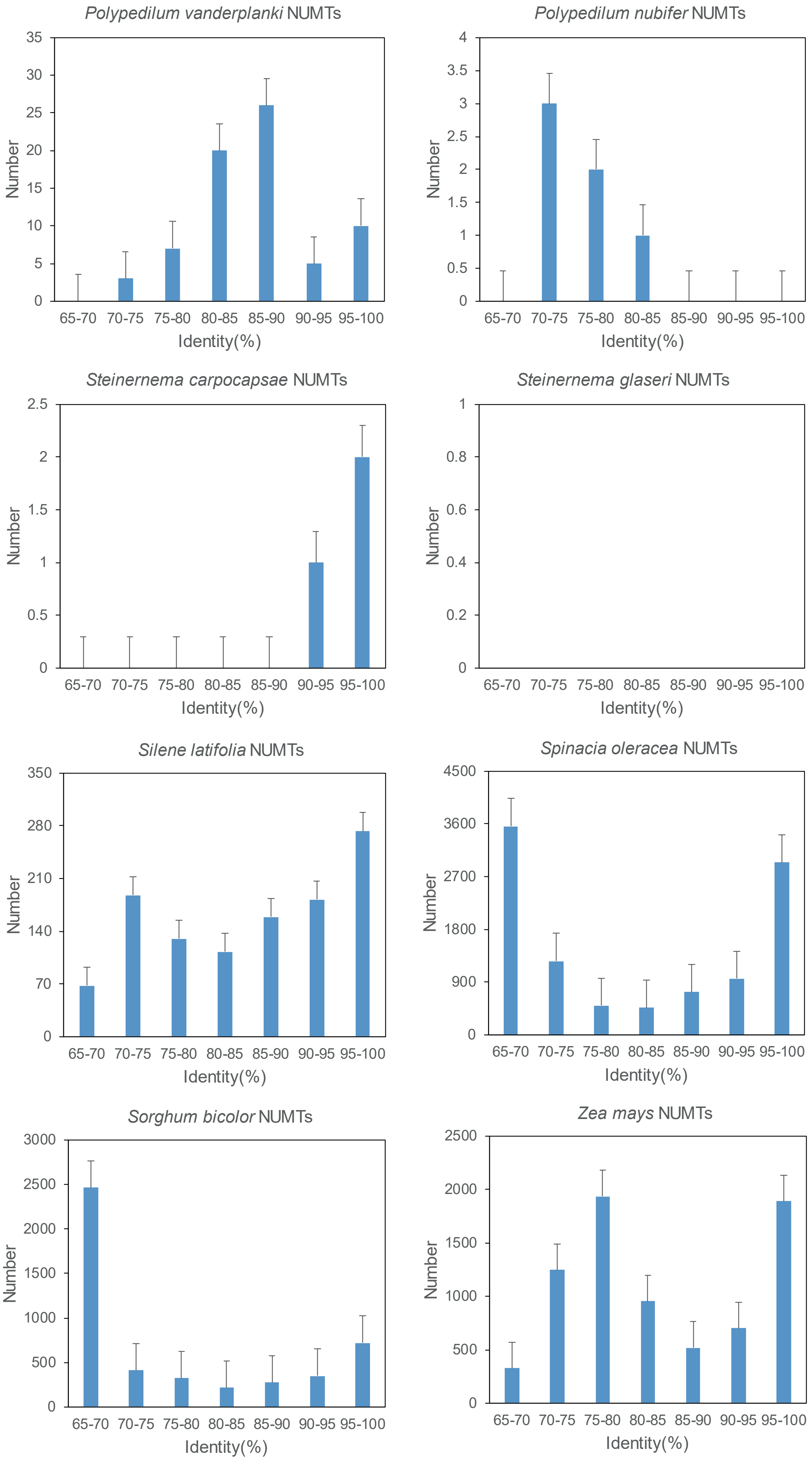

Continued on next page.

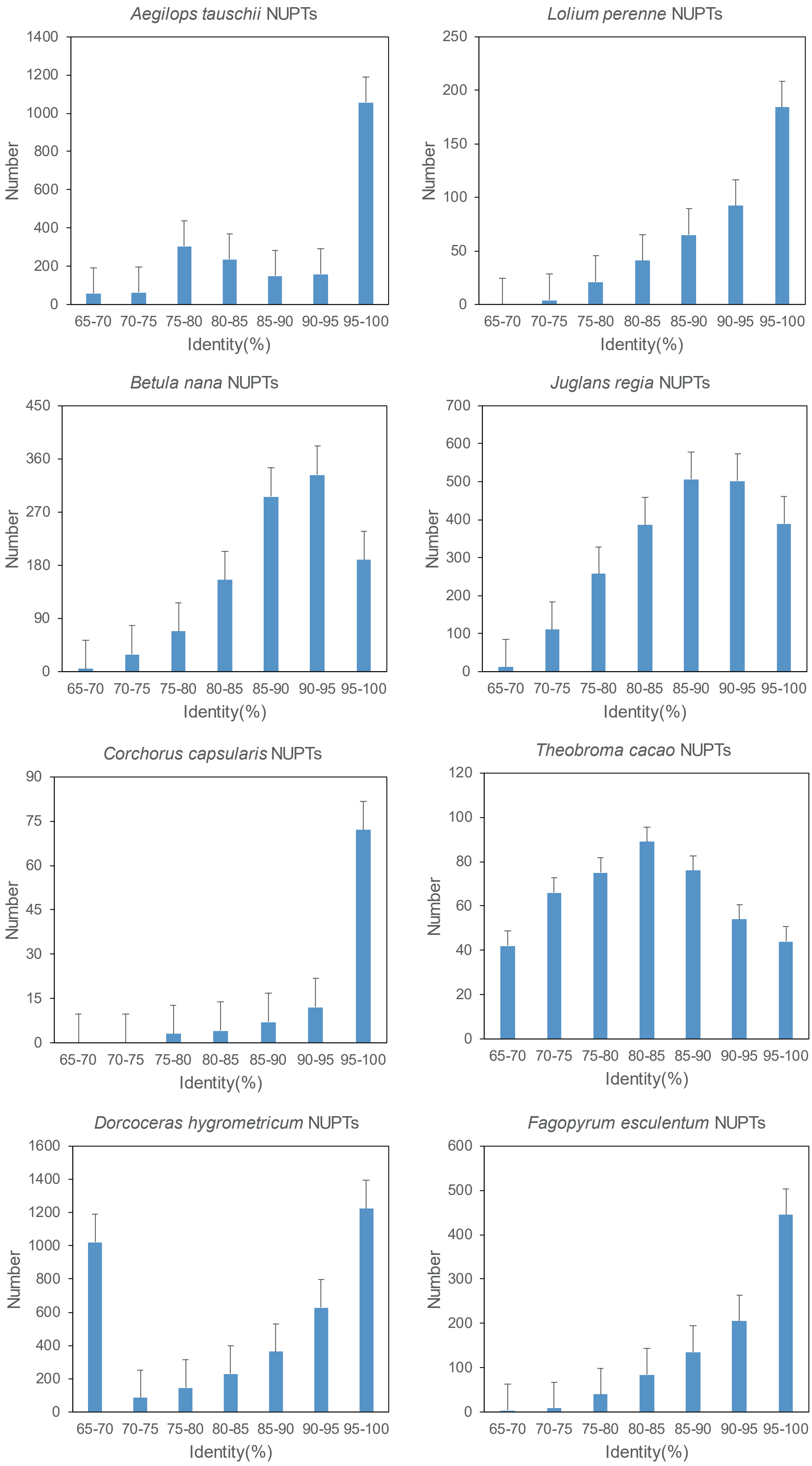

Continued on next page.

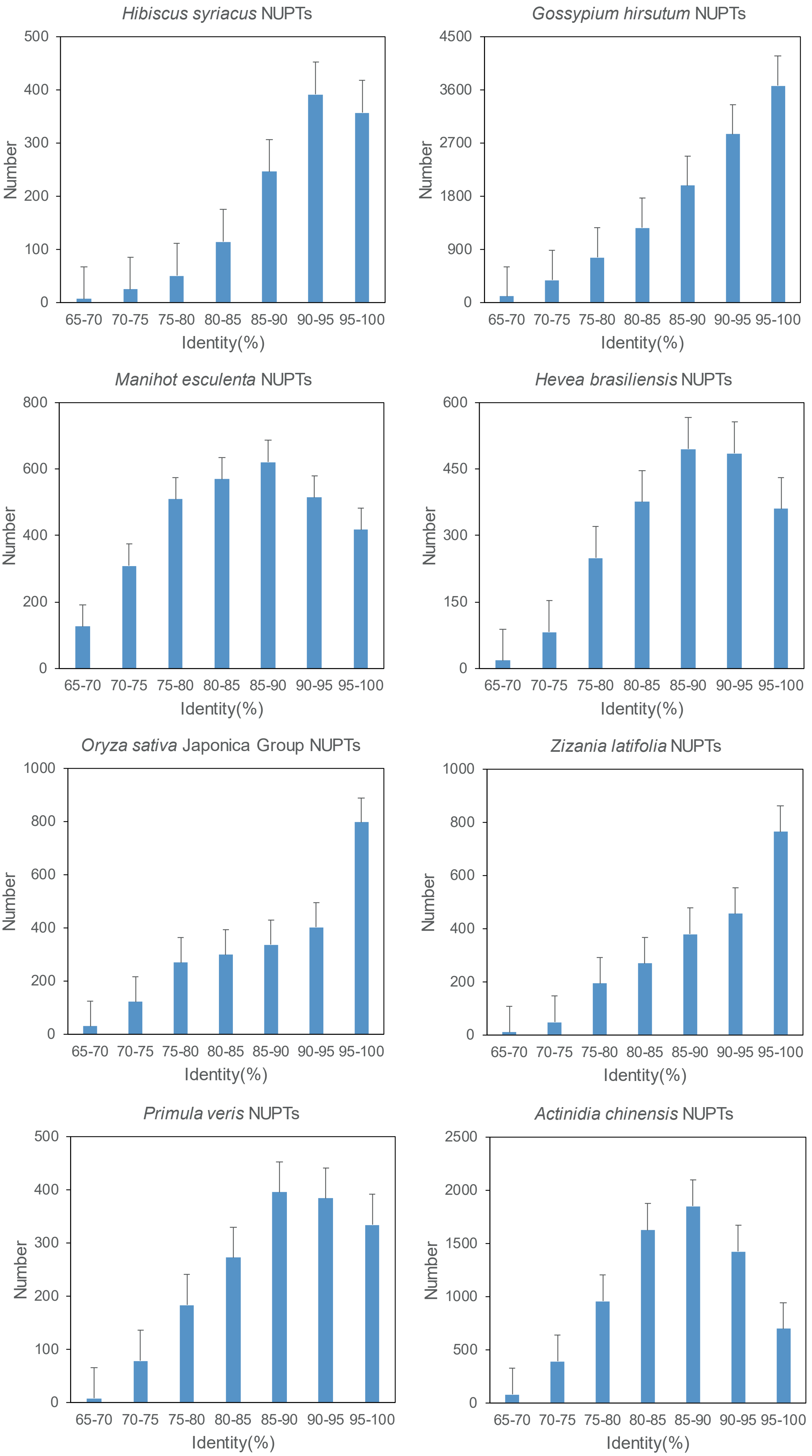

Continued on next page.

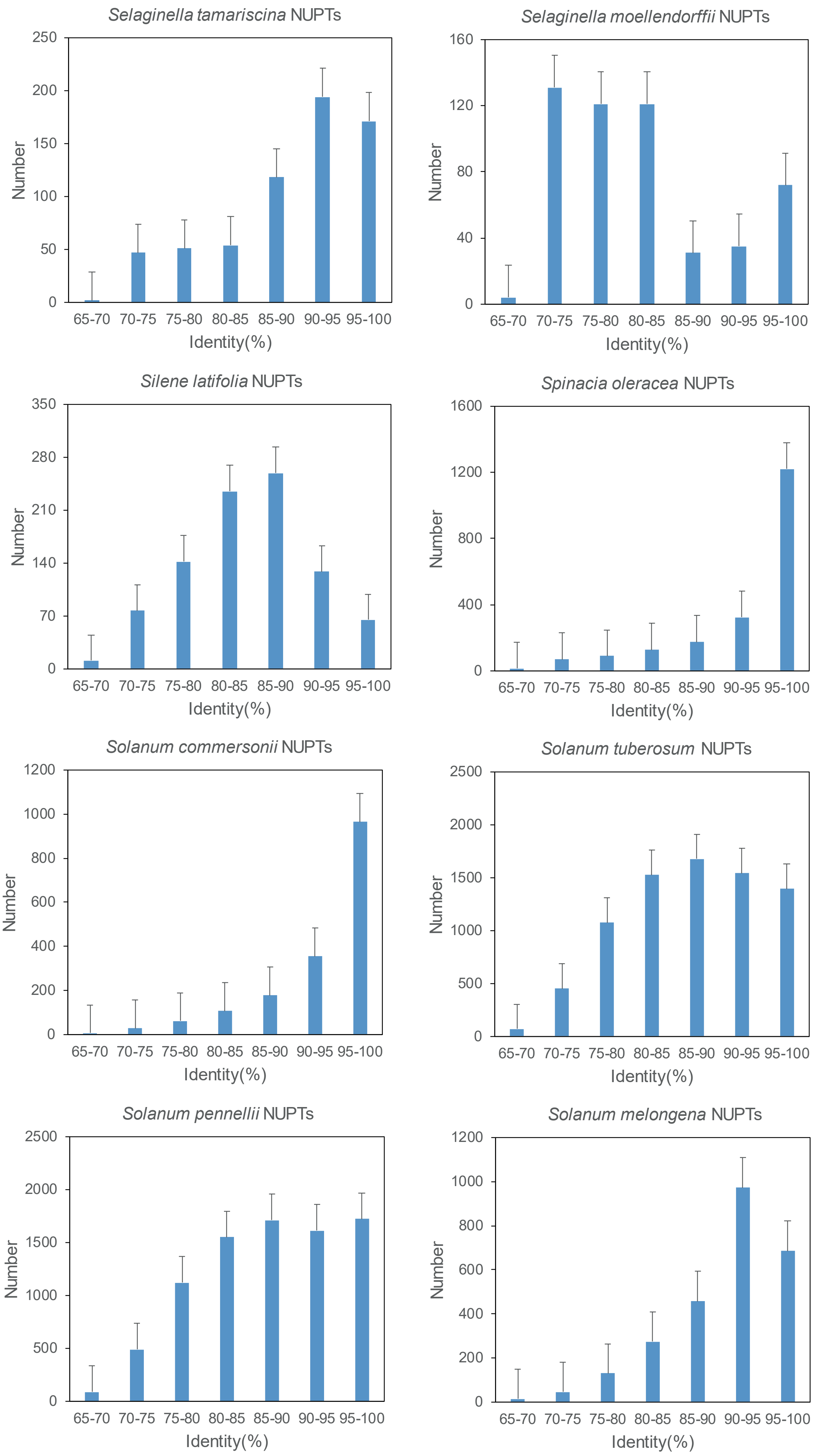

Continued on next page.

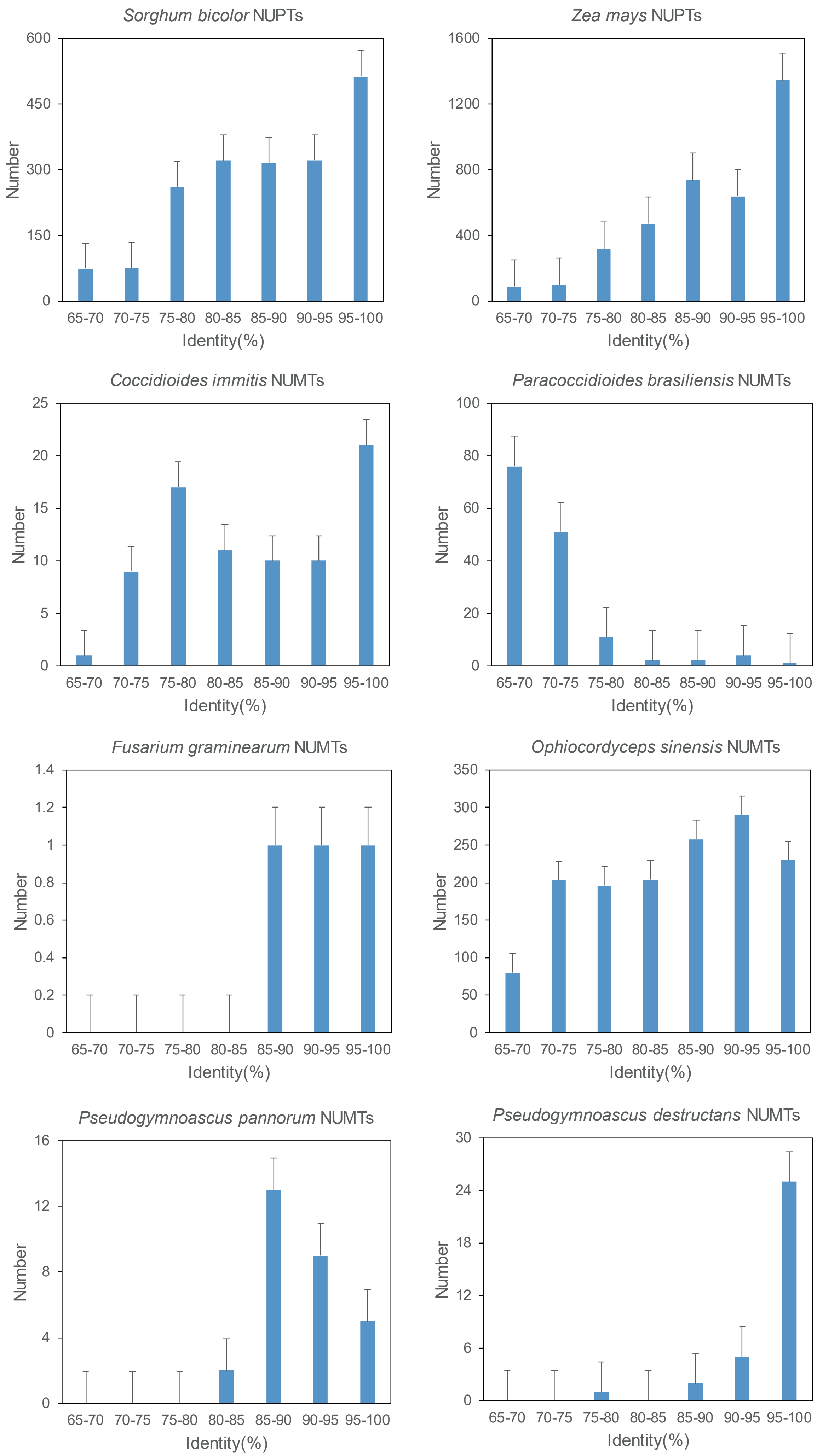
Sorghum bicolor NUPTs
Zea mays NUPTs
Coccidioides immitis NUMTs
Paracoccidioides brasiliensis NUMTs
Fusarium graminearum NUMTs
Ophiocordyceps sinensis NUMTs
Pseudogymnoascus pannorum NUMTs
Pseudogymnoascus destructans NUMTs
Number
Identity(%)
65-70
70-75
75-80
80-85
85-90
90-95
95-100
0
150
300
450
600
400
800
1200
1600
5
10
15
20
25
20
40
60
80
100
0.2
0.4
0.6
0.8
1
1.2
1.4
50
100
150
200
250
300
350
4
8
12
16
6
12
18
24
30
